# Bacterial Agents and Antimicrobial-Resistance Patterns in Canine Otitis Externa

**DOI:** 10.3390/ani15223317

**Published:** 2025-11-17

**Authors:** Sónia Saraiva, Rita Calouro, Telma de Sousa, Maria de Lurdes Enes Dapkevicius, João R. Mesquita, Ana C. Coelho, Patrícia Poeta

**Affiliations:** 1 Department of Veterinary Sciences, University of Trás-os-Montes and Alto Douro, 5000-801 Vila Real, Portugal; soniasaraiva@utad.pt (S.S.); accoelho@utad.pt (A.C.C.); 2 Animal and Veterinary Research Center (CECAV), University of Trás-os-Montes and Alto Douro, 5000-801 Vila Real, Portugal; ritasousacalouro@gmail.com; 3 Associate Laboratory of Animal and Veterinary Sciences (AL4AnimalS), 5000-801 Vila Real, Portugal; 4 Microbiology and Antibiotic Resistance Team (MicroART), University of Trás-os-Montes and Alto Douro, 5000-801 Vila Real, Portugal; telmaslsousa@hotmail.com; 5 Department of Genetics and Biotechnology, University of Trás-os-Montes and Alto Douro, 5000-801 Vila Real, Portugal; 6 Functional Genomics and Proteomics Unit, University of Trás-os-Montes and Alto Douro, 5000-801 Vila Real, Portugal; 7 Associated Laboratory for Green Chemistry, University NOVA of Lisbon, 1099-085 Caparica, Portugal; 8 Faculty of Agricultural and Environmental Sciences, University of the Azores, 9500-321 Angra do Heroísmo, Portugal; 9 Institute of Agricultural and Environmental Research and Technology (IITAA), University of the Azores, 9500-321 Angra do Heroísmo, Portugal; 10 School of Medicine and Biomedical Sciences (ICBAS), Porto University, 4050-313 Porto, Portugal; jrmesquita@icbas.up.pt

**Keywords:** antimicrobial resistance, dog, ear swab, ear culture, breed, recurrence

## Abstract

Canine otitis externa is a common and recurrent ear infection in dogs, often caused by bacterial pathogens, and complicated by increasing antimicrobial resistance. The analysis of bacterial isolates from dogs with otitis externa revealed a predominance of *Staphylococcus pseudintermedius*, representing 41% of all cases, followed by *Staphylococcus aureus* (23%), and *Pseudomonas aeruginosa* (19%). Other less frequently isolated organisms included *Escherichia coli*, *Streptococcus canis*, and *Proteus mirabilis*. These results highlight the significant role of *S. pseudintermedius* in the pathogenesis of otitis externa in dogs, as well as the relevance of Gram-negative opportunistic pathogens like *P. aeruginosa*, which exhibits the highest recurrence rate, with 90% of the cases associated with highly resistant to β-lactams (93% for amoxicillin–clavulanic acid; >70% for third-generation cephalosporins). *P. mirabilis* showed complete resistance to tetracycline, partial resistance to doxycycline, and reduced susceptibility to carbapenems, nitrofurantoin, and polymyxin B. *S. canis* exhibited limited resistance to erythromycin and clindamycin, while *S. epidermidis* displayed extensive multidrug resistance, including β-lactams, fluoroquinolones, sulfonamides, and polymyxins. These findings highlight the high prevalence of multidrug-resistant pathogens in canine otitis externa, emphasizing the need for culture-guided therapy and raising concerns regarding One Health, due to potential zoonotic transmission and dissemination of genetic determinants of antimicrobial resistance.

## Simple Summary

Otitis externa is among the most frequently diagnosed conditions in small animal veterinary practice, especially in dogs. It involves inflammation of the external ear canal and has a complex, multifactorial etiology. Contributing factors include breed-specific anatomical and physiological traits such as ear conformation and the density of ceruminous glands which can predispose certain dogs to the disease. Environmental influences, such as frequent exposure to moisture and outdoor activity, also play a role. Among pathogens, bacterial agents are of particular clinical importance, and increasing attention has been given to bacterial agents and resistance patterns in dogs with otitis externa, as antimicrobial resistance can significantly affect treatment outcomes. These interacting factors underscore the importance of a comprehensive diagnostic and therapeutic approach that considers both predisposing causes and emerging resistance trends in bacterial populations.

## 1. Introduction

Otitis externa is one of the most common diseases in small animal clinical practice, especially in dogs [1]. The estimated prevalence of canine otitis in Europe varies between 8.7% and 20% [2]. Despite its high prevalence, otitis externa in dogs is frequently underdiagnosed, particularly in rural or low-resource areas, due to geographic and socioeconomic constraints, diminished awareness of animal welfare, and a resulting low demand for veterinary services [3]. It is an inflammatory condition of the external ear canal with a complex and multifactorial etiology that involves anatomical predispositions, environmental factors, underlying diseases, and infectious agents [3,4]. Recent studies indicate that breeds such as Basset Hound, Chinese Shar Pei, Labradoodle, Beagle, Golden Retriever, Labrador Retriever, French Bulldog, and Yorkshire Terrier [5], as well as mixed-breed dogs [6], have a higher risk of otitis externa. Specific anatomical and physiological characteristics, including ear conformation and the density of ceruminous glands, contribute to this predisposition [7]. In affected dogs, the distribution of sebaceous and ceruminous glands remains similar to that in healthy ears; however, these glands become hyperplastic during otitis externa, and the hair follicles also exhibit hyperplasia [8]. Lifestyle factors, such as outdoor activities (e.g., swimming frequency), nutrition, and comorbidities, particularly atopy, are linked to changes in the ear microbiota that promote microbial growth [4]. Studies have identified a diverse range of bacteria associated with otitis externa, with the bacterial species involved varying between countries [9,10,11,12,13]. However, the bacterial agents most frequently isolated in cases of canine otitis externa include *Staphylococcus*, *Pseudomonas*, *Proteus*, and *Escherichia coli*. In Australia, the most prevalent isolates included *P. aeruginosa*, *Staphylococcus pseudintermedius*, streptococci, *Proteus* spp., and β-hemolytic *Streptococcus* spp. [12]. In France, the common pathogens identified included coagulase-positive staphylococci, *P. aeruginosa*, *P. mirabilis*, and *E. coli* [13]. Similarly, in Italy, *S. pseudintermedius*, *P. aeruginosa*, and *P. mirabilis* were among the most frequently identified bacteria [14]. A recently published report on canine otitis, encompassing 11 years, in the Iberian Peninsula, indicated that *Staphylococcus* (mainly *S. pseudintermedius*), *P. aeruginosa*, *Streptococcus* (*S. canis*), and *Enterobacterales* (*E. coli* and *P. mirabilis*) were the most frequently implicated genera/species, with prevalence of 35%, 20%, 13% and 11%, respectively [15].

Topical therapy is often effective in the short term; however, recurrent inflammatory or infectious episodes may progress to chronic otitis externa. Chronicity is characterized by structural alterations of the ear canal, persistent pain, and reduced responsiveness to treatment. Failure to accurately identify and manage the underlying etiology frequently results in prolonged antimicrobial use, thereby promoting the selection and proliferation of antimicrobial-resistant bacterial populations [2]. The empirical and repeated administration of antibiotics, without prior bacterial culture and antimicrobial susceptibility testing (AST), has substantially contributed to the emergence and dissemination of multidrug-resistant strains, including *Staphylococcus* spp. harboring the *mec* gene, which confers resistance to β-lactam antibiotics [2,16,17]. Antimicrobial resistance (AMR) currently represents a growing threat to global public health. Although historically associated with human medicine, AMR in companion animals has gained relevance, since these animals can act as reservoirs and vehicles for transmitting multidrug-resistant bacteria to the humans with whom they live [17,18]. In addition, the presence of AMR compromises therapeutic success, increases the costs associated with treatment, and negatively affects animal welfare [15]. In this context, monitoring bacterial resistance in clinical cases, in particular by systematically carrying out microbiological cultures and AST, is essential [18]. This approach makes it possible to guide more effective antimicrobial therapies, contribute to responsible antimicrobial stewardship programs in veterinary medicine, and therefore mitigate the impact of AMR on animal and human health [4], promoting the adoption of a true One Health approach to the mitigation of this fundamental threat to humankind.

In Portugal, although some studies have investigated canine otitis externa, particularly focusing on methicillin-resistant *S. aureus* (MRSA) and methicillin-resistant *S. pseudintermedius* (MRSP) [19,20], the available data remain limited and do not allow for meaningful comparisons with European trends or adequately support evidence-based clinical decision-making.

The present study aimed at characterizing the bacterial agents and AMR profiles isolated from cases of otitis externa in dogs presented to two veterinary clinics in Portugal (one in the northern region and one in the central region) and a hospital in the Lisbon region. In the present study, these profiles also enabled epidemiological comparisons and helped identify emerging resistance trends over the study period.

## 2. Materials and Methods

### 2.1. Sample Collection and Handling

Ear swab specimens were aseptically collected from 80 dogs clinically diagnosed with otitis externa. Samples were promptly transported to the laboratory for bacterial culture and antimicrobial susceptibility testing. All microbiological procedures were performed under strict aseptic conditions to ensure sample integrity. The analyzed specimens were collected over three years.

### 2.2. Criteria for the Selection of Dogs with Bacterial Otitis Externa

Dogs were enrolled based on well-defined clinical and microbiological criteria. Eligible cases presented clinical signs consistent with bacterial otitis externa, including erythema, pruritus, discharge, and malodor. The bacterial etiology was confirmed through culture and antimicrobial susceptibility testing. Cases associated with yeast infections, parasitic infestations, or secondary to systemic or dermatological disorders were excluded. Each case was subsequently classified as acute or chronic according to clinical history and recurrence. Acute otitis externa was defined as a first-episode or short-duration infection with rapid onset of clinical signs, whereas chronic cases were characterized by recurrent or persistent inflammation and a reduced response to therapy. Sampling was conducted continuously over the three-year study period (2023–2025).

### 2.3. Microbiological Analysis

Swabs were inoculated onto selective and differential media and incubated under appropriate conditions for bacterial growth. Colonies were isolated and identified using automated identification systems to confirm species-level classification.

### 2.4. Kirby–Bauer Disc Diffusion Assay

After bacterial agent identification, pure colonies were characterized according to their antibiotic resistance profiles using the Kirby–Bauer disk-diffusion method against 12 to 20 antibiotics (Oxoid, Thermo Fisher Scientific, Basingstoke, UK), following the standard protocol recommended by the European Committee on Antimicrobial Susceptibility Testing (EUCAST). Briefly, two to three colonies from overnight cultures were suspended in 1 mL of sterile 0.9% NaCl solution, and the turbidity was adjusted to match a 0.5 McFarland standard [21]. The standardized bacterial suspension was uniformly inoculated onto Mueller–Hinton agar plates (Oxoid, Hampshire, UK) using a sterile swab. Antibiotic discs (Oxoid, Basingstoke, UK) were placed on the agar surface at approximately 3 cm intervals, each containing a specific antimicrobial agent. The plates were incubated at 35 ± 1 °C for 18 ± 2 h, and the diameters of the inhibition zones were measured in millimeters (mm) and interpreted according to the EUCAST guidelines. In cases where EUCAST guidelines did not provide resistance criteria, the missing breakpoints were supplemented using the standards established by the Clinical and Laboratory Standards Institute (CLSI) [22]. Isolates were classified as resistant (R), susceptible with increased exposure (I), or susceptible with a standard dosing regimen (S) according to the interpretive categories and reference values defined by EUCAST [21].

### 2.5. Statistical Analyses

A log-linear regression model was applied to investigate associations between potential risk factors and the occurrence of canine otitis externa. Predictor variables included bacterial isolates, sex (male, female), age group (young, adult, geriatric), habitat (indoor, outdoor, mixed), and recurrence (present/absent). For each predictor, model estimates, standard errors, Z-values, and *p*-values were calculated. Model fit was assessed using deviance, the Akaike Information Criterion (AIC), and pseudo-R^2^ values. Multicollinearity and model assumptions were checked before analysis. The significance level (α) was set at 0.05, and *p*-values were compared to α to determine statistical significance. The statistical analyses were performed using JAMOVI version 2.3.28.3.

## 3. Results

**Figure 1:**
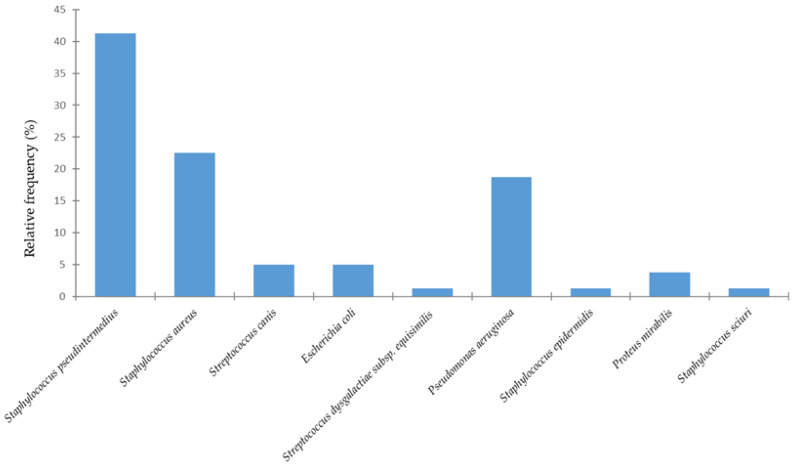
Distribution of bacterial species isolated from canine otitis externa.

Over the past three years (2023–2025), a total of 80 cases of canine otitis externa were recorded from two clinics and one veterinary hospital in Portugal, including 33 females and 47 males. Regarding breed distribution, Yorkshire Terriers (9%), Labradors (9%), and English Cocker Spaniels (7%) were the most commonly affected. Figure 1 shows the distribution of bacterial species isolated from dogs with otitis externa.

**Figure 2:**
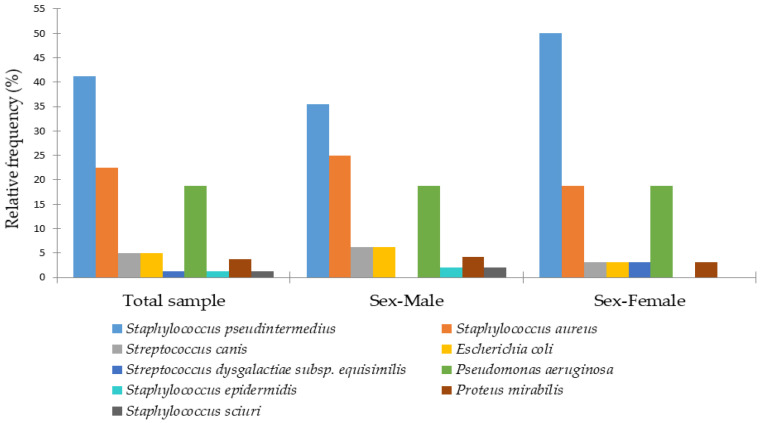
Distribution of bacterial species isolated from canine otitis externa in male and female dogs.

In the present study, the bacterial species most commonly associated with canine otitis externa was *S. pseudintermedius* (41%), followed by *S. aureus* (23%) and *P. aeruginosa* (19%). Less common isolates comprised *E. coli* (*n* = 4), *S. canis* (*n* = 4), *P. mirabilis* (*n* = 3), besides single occurrences of other bacterial species. Figure 2 illustrates the relative frequencies of female and male dogs according to the bacterial etiology of otitis externa.

Analysis by sex revealed that *S. pseudintermedius* was more frequently detected in females (50%) than in males (35%). Males exhibited a greater diversity of less common bacterial species, such as *Staphylococcus sciuri* and *Staphylococcus epidermidis*, which were absent in females. *S. aureus* was more prevalent in males (25%) compared to females (19%), whereas *P. aeruginosa* occurred at similar frequencies in both sexes (18.8%) (Figure 2).

**Figure 3:**
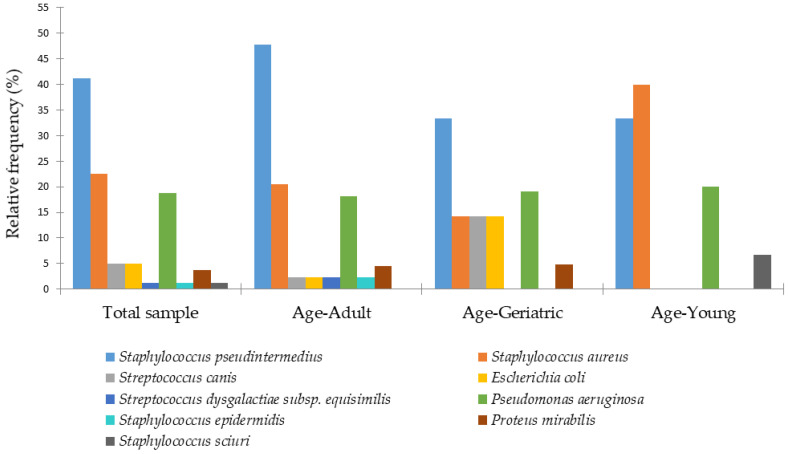
Distribution of bacterial species isolated from canine otitis externa across different age groups.

The distribution of otitis externa according to age groups (young/young adult, adult, and geriatric) is presented in Figure 3, highlighting potential age-related patterns in bacterial prevalence.

**Figure 4:**
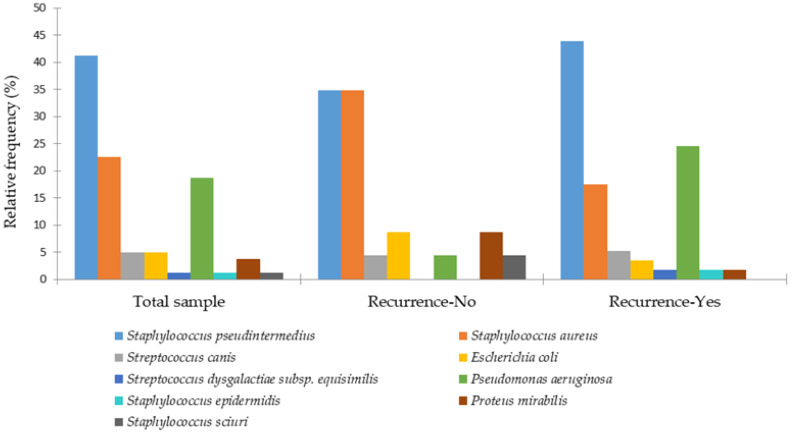
Distribution of bacterial species isolated from canine otitis externa by recurrence.

In young dogs, otitis was mainly associated with *S. aureus* (40%) and *S. pseudintermedius* (33%), whereas in adult dogs *S. pseudintermedius* was predominant (48%). In geriatric dogs, bacterial otitis externa was most commonly caused by *S. pseudintermedius* (33%), followed by *P. aeruginosa* (19%), with a notably higher proportion of *E. coli* (14%) compared to the other age groups. Figure 4 shows the frequency of recurrence of otitis externa by bacterial etiology.

The bacterial pathogens most commonly associated with non-recurrent otitis externa in dogs were *S. aureus* (35% vs. 18%), *P. mirabilis* (9% vs. 2%), and *E. coli* (9% vs. 4%). In contrast, recurrent otitis externa was more frequently associated with *P. aeruginosa* infections (25% vs. 4%).

**Figure 5:**
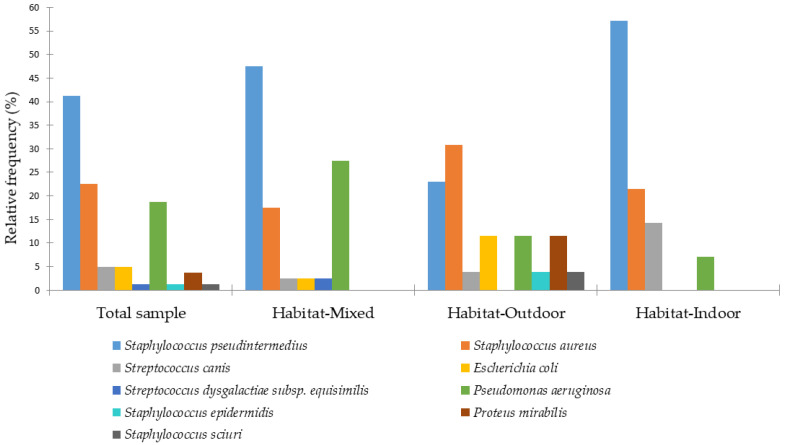
Distribution of bacterial species isolated from canine otitis externa according to the dog’s habitat.

The distribution of otitis externa among dogs is shown based on their habitat (indoor, outdoor, or mixed) in Figure 5.

In outdoor dogs, *S. aureus* was the most frequent cause of otitis externa (31%), while *S. pseudintermedius* predominated in indoor (57%) and mixed habitats (48%). All cases of *P. mirabilis* (12%) and a higher proportion of *E. coli* (12%) were found in outdoor dogs. *P. aeruginosa* reached its highest prevalence in mixed habitats (28%), and *S. aureus* was the second most common isolate in indoor dogs (21%). Table 1 presents the results of a log-linear regression analysis examining factors associated with otitis externa in dogs.

The log-linear regression model showed a good fit to the data, explaining approximately 64.4% of the total variation (R^2^ = 0.644). The intercept (estimate = 1.96, *p* < 0.001) represents the expected log rate of otitis externa for the reference group, which includes males with no recurrence and living in a mixed habitat. Exponentiating this value, the expected rate of otitis externa is approximately 7.1. Sex had a significant effect on the outcome (*p* = 0.046). Females had a lower expected rate of otitis externa compared to males, with a rate ratio of 0.63, indicating that females experienced approximately 37% fewer cases than males, controlling for other factors. Dogs with a history of otitis externa have a 2.6 times higher expected rate of experiencing a new episode compared to dogs without such a history, controlling for sex and habitat.

Habitat also had a significant effect. Compared to individuals in mixed habitats, those in outdoor habitats had an expected rate roughly 50% lower (rate ratio = 0.50, *p* = 0.009), while those in indoor habitats had an expected rate about 60% lower (rate ratio = 0.40, *p* = 0.002). Thus, individuals in mixed environments exhibited the highest expected rates of otitis externa, followed by those in outdoor and indoor habitats.

Overall, the results indicate that recurrence substantially increases the expected rate of otitis externa, while female sex and living in indoor or outdoor habitats (as opposed to mixed environments) are associated with lower expected rates.

The majority of *S. pseudintermedius* isolates exhibited high resistance to β-lactams, including amoxicillin (55%), amoxicillin–clavulanic acid (38%), ampicillin (61%), penicillin G (69%), and methicillin (35%). Resistance to clindamycin was 27%, followed by enrofloxacin at 9%. A similar resistance profile, but with higher overall levels, was observed for *S. aureus* against β-lactams, including amoxicillin (69%), amoxicillin–clavulanic acid (47%), ampicillin (67%), and penicillin G (72%). Moreover, 28% of the isolates were positive for the *mecA* gene. Resistance was also detected to clindamycin (38%), azithromycin (22%), and marbofloxacin (17%). Antimicrobial susceptibility testing of *E. coli* isolates revealed that 50% were ESBL-positive and exhibited resistance to β-lactams, including amoxicillin, amoxicillin–clavulanic acid, ampicillin, cefadroxil, cephalexin, cephalothin, cefovecin, cefpodoxime, and ceftiofur, as well as to the fluoroquinolones (enrofloxacin, marbofloxacin, and pradofloxacin) and to doxycycline and chloramphenicol. *P. aeruginosa* exhibited high-level resistance to β-lactams, including amoxicillin–clavulanic acid (93%) and third-generation cephalosporins, cefovecin (86%) and ceftiofur (73%).

All *P. mirabilis* isolates were resistant to tetracyclines, with 100% resistance to tetracycline and 67% to doxycycline. Resistance to carbapenems was also observed, with 33% of isolates resistant and 67% exhibiting intermediate susceptibility to imipenem. Additionally, 33% of *P. mirabilis* isolates showed resistance to nitrofurantoin, ampicillin, cephalexin, and polymyxin B. In the case of otitis caused by *S. canis*, resistance was observed only to erythromycin (25%) and clindamycin (25%). No multidrug resistance profiles were detected. *S. epidermidis* exhibited multidrug resistance, including resistance to penicillin G, sulfamethoxazole–trimethoprim, polymyxin B, and fluoroquinolones (enrofloxacin, marbofloxacin, and pradofloxacin).

## 4. Discussion

Otitis externa, a common condition in canine veterinary medicine, is influenced by several risk factors, including breed, body weight relative to the breed–sex mean, age, sex, and insurance status [5]. In the present study, Yorkshire Terriers (9%), Labradors (9%), and English Cocker Spaniels (7%) exhibited the highest frequencies of otitis externa. Strong breed-related effects as predisposing factors have been reported in other studies, with dogs exhibiting pendulous or V-shaped drop pinnae showing a higher risk of developing otitis externa compared to those with erect pinnae [2,23]. Age, the amount of hair in the external ear canal, and ambient temperature are also predisposing factors for otitis externa [24]. A better understanding of predisposing factors in high-risk dogs should be further explored, as it can contribute to earlier detection of otitis externa cases and to improved owner compliance with preventive and therapeutic measures.

In the present study, the bacteria most commonly associated with otitis externa in dogs were *S. pseudintermedius* (41%), followed by *S. aureus* (23%) and *P. aeruginosa* (19%), indicating that these are the primary bacterial agents involved in this condition. These findings are broadly consistent with previous reports; for instance, in Serbia, *S. pseudintermedius* accounted for 65.8% of isolates while *S. aureus* represented 22.4% [25].

*S. pseudintermedius* is a significant pathogenic bacterium in veterinary medicine, causing a variety of infections [25]. *S. pseudintermedius* is a pathogenic bacterium of concern within the veterinary sector and is involved in numerous infections in canines, including topical infections such as canine pyoderma and otitis externa, as well as systemic infections within the urinary, respiratory and reproductive tract. Studies report that *S. pseudintermedius* is a predominant pathogen associated with canine otitis externa, isolated from 20–94.3% of otitis externa cases in dogs [26,27,28]. Its zoonotic potential has been highlighted in a recent study that compared *S. pseudintermedius* carriage in dogs and their tutors [29]. In the present study, the majority of *S. pseudintermedius* isolates exhibited high resistance to β-lactams, including amoxicillin (55%), amoxicillin–clavulanic acid (38%), ampicillin (61%), penicillin G (69%), and methicillin (35%). In a recent study conducted in Romania, Dégi et al. [30] assessed the antibiotic susceptibility patterns of *Staphylococcus* species isolated from canine otitis externa cases. Their findings revealed a 69% resistance rate to Penicillin G, aligning with the results of the present study and supporting previously reported evidence of decreased efficacy of β-lactam antibiotics against *Staphylococcus* isolates from companion animals. The high prevalence of methicillin-resistant *S. pseudintermedius* (MRSP) in such infections is a growing concern globally. β-lactam antibiotics are often the first-line treatment for *Staphylococcus*-associated infections [30] and resistance to these drugs poses significant therapeutic challenges in veterinary medicine [31,32]. Resistance to lincosamides was also observed (clindamycin, 27%), followed by fluoroquinolones (enrofloxacin, 9%). A similar resistance profile, but with higher overall levels, was observed for *S. aureus* against β-lactams, including amoxicillin (69%), amoxicillin–clavulanic acid (47%), ampicillin (67%), and penicillin G (72%). Additionally, 28% of the isolates carried the *mecA* gene. The production of β-lactamase by *S. aureus* is the primary mechanism underlying resistance to penicillin and its derivatives. In contrast, methicillin resistance is mediated by the *mecA* gene, which encodes an altered penicillin-binding protein (PBP2a). The *mecA* gene, responsible for methicillin resistance, is located on the MRSA chromosome, and, to date, seven distinct staphylococcal chromosomal cassette mec (SCCmec) types have been identified [33]. In the first report from Cyprus on methicillin resistance in dogs and cats, *S. pseudintermedius* and *S. aureus* isolates were analyzed. PCR analysis revealed that 10% of isolates were MRSP and 43% were MRSA [34]. Resistance in *S. aureus* isolates was also detected against lincosamides (clindamycin, 38%), macrolides (azithromycin, 22%), and fluoroquinolones (marbofloxacin, 17%). Beyond β-lactams, resistance to clindamycin (38%) and azithromycin (22%) further narrows the spectrum of effective treatments, especially since macrolides and lincosamides are sometimes used as alternatives in skin and ear infections. The detection of fluoroquinolone resistance (17% to marbofloxacin) is also noteworthy, given that fluoroquinolones are commonly used in veterinary dermatology and otitis management; thus, resistance at this level may compromise empirical therapy and accelerate the spread of multidrug-resistant strains.

Antimicrobial susceptibility testing of *E. coli* isolates revealed that 50% were ESBL-positive, exhibiting resistance to multiple β-lactams—including amoxicillin, amoxicillin–clavulanic acid, ampicillin, cefadroxil, cephalexin, cephalothin, cefovecin, cefpodoxime, and ceftiofur—as well as to fluoroquinolones (enrofloxacin, marbofloxacin, pradofloxacin), doxycycline, and chloramphenicol. In a study conducted in Greece, *E. coli* isolated from dogs with otitis externa exhibited high levels of fluoroquinolone resistance. The predominant mechanism of resistance to enrofloxacin, marbofloxacin, and pradofloxacin was associated with mutations in topoisomerase genes [35]. In the present study, *P. aeruginosa* was the third most commonly isolated bacterium associated with otitis externa in dogs, with a prevalence of 19%, in line with the 20% prevalence value found in a recent Iberian study [15], and consistent with its frequent isolation from canine otitis cases worldwide [36,37]. For instance, another study in northern Portugal analyzed 236 *P. aeruginosa* samples; the majority of them were associated with otitis in dogs and cats. Resistance to fluoroquinolones such as enrofloxacin (25 isolates) and marbofloxacin (38 isolates) was also significant [38]. In the present study, *P. aeruginosa* isolates exhibited extremely high levels of resistance to β-lactams, with amoxicillin–clavulanic acid ineffective in 93% of cases, and third-generation cephalosporins such as cefovecin and ceftiofur failing in 86% and 73% of cases, respectively. Consistent with the present findings, a study from Portugal documented markedly elevated resistance rates to cephalosporins, including ceftiofur and cefovecin, in bacterial isolates obtained from dogs [38]. These findings highlight the formidable challenge posed by this pathogen and underscore the urgent need for culture-guided therapy. Other studies investigating the resistance profile of *P. aeruginosa* have identified ciprofloxacin as the most effective drug to treat infections by this bacterial pathogen. However, these studies also report alarming levels of resistance among *P. aeruginosa* isolates from canine otitis externa samples [36,37]. *P. aeruginosa* is not a typical constituent of the normal canine ear microbiota, but it is frequently isolated from cases of chronic otitis externa [39]. *P. aeruginosa* is a frequent pathogen in chronic suppurative otitis externa, which often develops as a long-term progression of persistent or recurrent otitis that can last for months or even years. This type of otitis is typically characterized by the clinical presence of whitish, viscous ear exudate [40]. Its pathogenic nature often complicates treatment. Biofilm formation has been observed in 40–95% of *P. aeruginosa* isolates from otitis externa cases, and both intrinsic and acquired resistance to clinically important antibiotics further underscores the challenges of managing this infection [39]. This bacterium produces β-lactamases, including AmpC cephalosporinases and extended-spectrum β-lactamases (ESBLs), which hydrolyze penicillins, aminopenicillins, and many cephalosporins. Moreover, *P. aeruginosa* possesses multiple efflux systems (e.g., MexAB-OprM) that actively expel β-lactams, fluoroquinolones, and aminoglycosides, contributing to multidrug resistance [39]. The striking resistance rates reported in our study and in other works, such as Garcias et al. [15] underscore the formidable challenge this pathogen poses to treatment and highlights the critical importance of targeted, culture-guided antimicrobial therapy. Overall, these results indicate that *S. pseudintermedius* and *P. aeruginosa* are the primary bacterial drivers of otitis externa in the studied population. *S. pseudintermedius* appears particularly dominant across multiple habitats and in both sexes, highlighting its central role in the etiology of the disease. Meanwhile, *P. aeruginosa* shows a notable prevalence, especially in dogs from mixed habitats, suggesting that environmental factors may influence its distribution. These findings emphasize the importance of targeted diagnostic testing and appropriate antimicrobial therapy, particularly against these key pathogens, to effectively manage and prevent otitis externa. They also emphasize the importance of bacterial agents involved in canine otitis as potential zoonotic pathogens, as well as the need to implement effective One Health-based measures to mitigate the threats associated with these organisms and their genetic determinants of AMR.

## 5. Conclusions

*S. pseudintermedius*, *S. aureus*, and *P. aeruginosa* were the main bacterial pathogens associated with otitis externa in dogs, and their prevalence was influenced by ear conformation and habitat. High levels of antimicrobial resistance—particularly methicillin resistance in staphylococci and multidrug resistance in *P. aeruginosa*—underscore the importance of performing routine culture and susceptibility testing before prescribing antibiotics, especially for recurrent or chronic cases. Empirical use of β-lactams for staphylococcal infections should be avoided in Portugal due to high resistance rates to this class of antibiotics. This information is useful for veterinarians in the region where the study took place, aiding clinicians to make informed decisions when treating bacterial canine otitis. These results also emphasize potential One Health implications, as resistant pathogens in companion animals may pose a zoonotic risk to humans.

## Figures and Tables

**Table 1:** Log-linear Regression Model for Canine Otitis Externa: Estimates, Standard Errors, Z-values, and *p*-values.

Predictor	Estimates	95% Confidence Interval		SE	Z	*p*	Rate Ratio (Exp(Estimate))
		Lower	Upper				
Intercept	1.96	1.46	2.45	0.25	7.69	<0.001	7.07
Sex							
Female–Male	−0.46	−0.91	−0.01	0.23	−1.99	0.046	0.63
Recurrence							
Yes–No	0.97	0.48	1.46	0.25	3.87	<0.001	2.64
Habitat							
Outdoor–Mixed	−0.69	−1.22	−0.17	0.27	−2.59	0.009	0.50
Indoor–Mixed	−0.90	−1.47	−0.34	0.29	−3.15	0.002	0.40

Note: Reference categories: Male (Sex), No recurrence (Recurrence), Mixed habitat (Habitat).

## Data Availability

The data supporting the findings of this study are available from the corresponding authors upon reasonable request.
